# The performance of the practices associated with the occurrence of severe intraventricular hemorrhage in the very premature infants: data analysis from the Chinese neonatal network

**DOI:** 10.1186/s12887-024-04664-8

**Published:** 2024-06-14

**Authors:** Tiantian Xiao, Liyuan Hu, Huiyao Chen, Xinyue Gu, Jianguo Zhou, Yanping Zhu, Xiaoping Lei, Siyuan Jiang, Yulan Lu, Xinran Dong, Lizhong Du, Shoo K. Lee, Rong Ju, Wenhao Zhou, for the Chinese Neonatal Network, Lin Yuan, Tongling Yang, Hao Yuan, Li Wang, Chao Chen, Yun Cao, Xiuyong Chen, Huyan Zhang, Xiuying Tian, Jingyun Shi, Zhankui Li, Chuanzhong Yang, Ling Liu, Zuming Yang, Jianhua Fu, Yong Ji, Dongmei Chen, Changyi Yang, Rui Chen, Xiaoming Peng, Ruobing Shan, Shuping Han, Hui Wu, Lili Wang, Qiufen Wei, Mingxia Li, Yiheng Dai, Hong Jiang, Wenqing Kang, Xiaohui Gong, Xiaoyun Zhong, Yuan Shi, Shanyu Jiang, Bing Sun, Long Li, Zhenlang Lin, Jiangqin Liu, Jiahua Pan, Hongping Xia, Xiaoying Li, Falin Xu, Yinping Qiu, Li Ma, Ling Yang, Xiaori He, Yanhong Li, Deyi Zhuang, Qin Zhang, Wenbin Dong, Jianhua Sun, Kun Liang, Huaiyan Wang, Jinxing Feng, Liping Chen, Xinzhu Lin, Chunming Jiang, Chuan Nie, Linkong Zeng, Mingyan Hei, Hongdan Zhu, Hongying Mi, Zhaoqing Yin, Hongxia Song, Hongyun Wang, Dong Li, Yan Gao, Yajuan Wang, Liying Dai, Liyan Zhang, Yangfang Li, Qianshen Zhang, Guofang Ding, Jimei Wang, Xiaoxia Chen, Zhen Wang, Zheng Tang, Xiaolu Ma, Xiaomei Zhang, Xiaolan Zhang, Fang Wu, Yanxiang Chen, Ying Wu

**Affiliations:** 1 Department of Neonatology, School of Medicine, Chengdu Women’s and Children’s Central Hospital, School of Medicine, University of Electronic Science and Technology of China, Chengdu, China; 2 Department of Neonatology, NHC Key Laboratory of Neonatal Diseases, Fudan University, Children’s Hospital of Fudan University, Shanghai, China; 3 Center for Molecular Medicine, Children’s Hospital of Fudan University, Shanghai, China; 4 NHC Key Laboratory of Neonatal Diseases, Children’s Hospital of Fudan University, Shanghai, China; 5 Department of Neonatology, First Affiliated Hospital of Xinjiang Medical University, Urumqi, Xinjiang China; 6 Division of Neonatology, Department of Pediatrics, the Affiliated Hospital of Southwest Medical University, Luzhou, Sichuan China; 7 Neonatal Intensive Care Unit, Children’s Hospital, Zhejiang University School of Medicine, Hangzhou, China; 8 National Clinical Research Center for Child Health, National Children’s Regional Medical Center, Hangzhou, China; 9 Maternal-Infant Care Research Centre and Department of Pediatrics, Mount Sinai Hospital, Toronto, ON Canada

**Keywords:** Prematurity, Severe intraventricular hemorrhage, Practice, Multiple neonatal intensive care units

## Abstract

**Background:**

The occurrence of severe intraventricular hemorrhage (sIVH) was high in the very preterm infants (VPIs) in China. The management strategies significantly contributed to the occurrence of sIVH in VPIs. However, the status of the perinatal strategies associated with sIVH for VPIs was rarely described across the multiple neonatal intensive care units (NICUs) in China. We aim to investigate the characteristics of the perinatal strategies associated with sIVH for VPIs across the multiple NICUs in China.

**Methods:**

This was a retrospective analysis of data from a prospective cohort of Chinese Neonatal Network (CHNN) dataset, enrolling infants born at 24^+0^—31^+6^ from 2019 to 2021. Eleven perinatal practices performed within the first 3 days of life were investigated including antenatal corticosteroids use, antenatal magnesium sulphate therapy, intubation at birth, placental transfusion, need for advanced resuscitation, initial inhaled gas of 100% FiO2 in delivery room, initial invasive respiratory support, surfactant and caffeine administration, early enteral feeding, and inotropes use. The performances of these practices across the multiple NICUs were investigated using the standard deviations of differences between expected probabilities and observations. The occurrence of sIVH were compared among the NICUs.

**Results:**

A total of 24,226 infants from 55 NICUs with a mean (SD) gestational age of 29.5 (1.76) and mean (SD) birthweight of 1.31(0.32) were included. sIVH was detected in 5.1% of VPIs. The rate of the antenatal corticosteroids, MgSO4 therapy, and caffeine was 80.0%, 56.4%, and 31.5%, respectively. We observed significant relationships between sIVH and intubation at birth (AOR 1.52, 95% CI 1.13 to 1.75) and initial invasive respiratory support (AOR 2.47, 95% CI 2.15 to 2.83). The lower occurrence of sIVH (4.8%) was observed corresponding with the highest utility of standard antenatal care, the lowest utility of invasive practices, and early enteral feeding administration.

**Conclusions:**

The current evidence-based practices were not performed in each VPI as expected among the studied Chinese NICUs. The higher utility of the invasive practices could be related to the occurrence of sIVH.

**Supplementary Information:**

The online version contains supplementary material available at 10.1186/s12887-024-04664-8.

## Introduction

Intraventricular hemorrhage (IVH) is one of the serious threats to survival for preterm infants [1]. Severe intraventricular hemorrhage (sIVH) has historically been defined as greater than or equal to grade 3 according to the Papile criteria [2]. The incidence of sIVH was approximately 11.6% (19,781/170, 031) in very preterm infants (VPIs) globally [3]. China has the second largest number of preterm infants globally [4]. Recent data from the Chinese Neonatal Network (CHNN) reported that 10.4% (745/7,189) of the VPIs were diagnosed with sIVH or cystic periventricular leukomalacia [5]. The overall trend of sIVH is decreasing with improvement of neonatal care, but the rates of sIVH are still relatively high in VPIs globally [3, 6–11].

The presence of sIVH has been strongly associated with the adverse long-term neurodevelopmental outcomes [12]. Additionally, the sIVH was one of the major reasons for discharge against medical advice (DAMA) among VPIs in China [13, 14]. Most of sIVH was diagnosed within 7 days of life (DOL) [15], and no specific therapy exists to treat the sIVH after it has occurred. Therefore, the prevention of sIVH is crucial but challenging in this narrow window of time.

The management strategies contributing to the occurrence of sIVH [16], include outborn neonatal transport [17], handling and minimizing elevations of blood pressure, midline head positioning etc.. Studies have demonstrated potentially beneficial practices for the prevention of sIVH in preterm infants include improved antenatal corticosteroid use, early noninvasive ventilation, delayed cord clamping, and risk-based indomethacin prophylaxis [18–22]. Other studies suggest that there is lower mortality of VPIs at 2 years or better neurological outcomes in the NICUs with higher proportions of free of mechanical ventilation at 24 h of life, early enteral feeding, and consistent neurodevelopmental care practices [3, 23].

In China, there is a large number of NICUs [24], therefore, the practice variations can be quite substantial across the different units [24]. As stated above [5, 14], decreasing the occurrence of sIVH in China is still a big challenge, and quality improvement projects aimed at reducing the number of sIVH in VPIs is necessary. Nonadherence to evidence based best practices may contribute to adverse outcomes in VPIs. However, little data exists to systematically describe the practice differences across Chinese NICUs. After reviewing a systematic review and meta-analysis [25], we planned to analyze common perinatal practices associated with sIVH or preterm mortality which were available in the CHNN dataset. Therefore, our main objective was to characterize the performance of the perinatal practices including antenatal corticosteroids use, antenatal magnesium sulphate (MgSO4) therapy, intubation at birth, placental transfusion, need for advanced resuscitation, initial inhaled gas of 100% FiO2 in delivery room, initial invasive respiratory support, surfactant and caffeine administration, early enteral feeding administration, and inotropes use which are associated with sIVH based on data from the Chinese Neonatal Network.

## Method

### Population and data source

A retrospective, hospital-based cohort of all infants born at gestational age of 24^+0^—31^+6^ were derived from the CHNN database between January, 1st, 2019, through December, 31st, 2021. This cohort included 77 participating Chinese children’s or maternal and children’s hospitals where the levels of NICUs were level III [5]. The CHNN view board approved the study and waived consent.

**Fig. 1:**
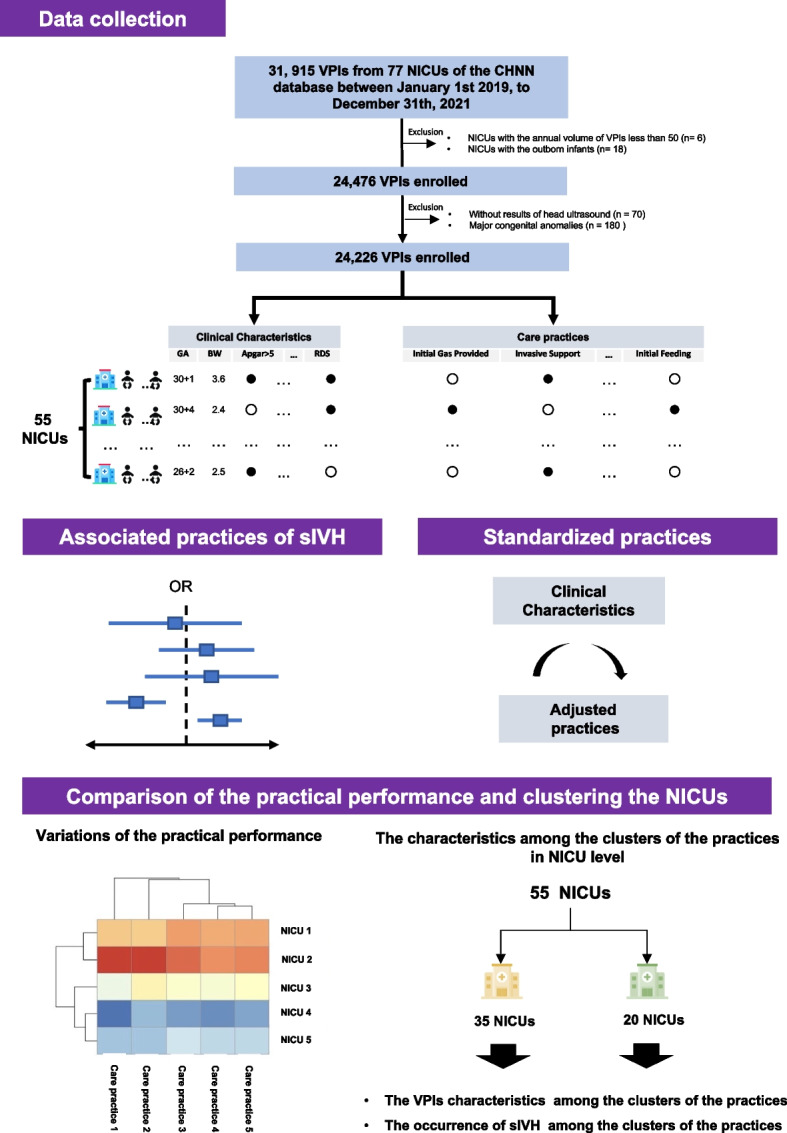
The workflow of this study

To prevent referral and recalled bias because of the uncertainty of the clinical practice in the delivery room of referring hospital, we only included infants who were inborn and admitted into NICU within 24 h of life and the data of each infant was complete. Considering the annual volume of the VPIs associated with death or sIVH [26], we only included the NICUs which had an average 50 VPI admissions per year or more in order to limit the bias of experience and ability of hospitals. Exclusion criteria were infants with major congenital anomalies, and infants without the results of head ultrasound (Fig. 1).

### Data collection and datasets

The CHNN database contains a record of maternal demographics, procedures, medication administration, nutrition and respiratory support for each day of an infant’s hospitalization. The initial head ultrasound was routinely performed after day 3 of life, and sIVH is diagnosed mostly within 4–7 days of life [15, 27]. Therefore, we focused primarily on clinical practices in the first 3 DOL.

### The classification of early perinatal practices and definitions

After reviewing a systematic review and meta-analysis [25], we planned to analyze common perinatal practices associated with sIVH or preterm mortality which were available in the CHNN dataset. We extracted eleven practice variables including antenatal corticosteroid use, antenatal MgSO4 therapy, intubation at birth, placenta transfusion, advanced resuscitation, initial inhaled gas of 100% FiO2 in delivery room, initial invasive respiratory support, surfactant administration, treatment with caffeine within 3 DOL, enteral feeding administration within 3 DOL, and inotropes usage. The definitions of the above early perinatal practices were described in Supplemental materials.

### The perinatal clinical characteristics and definitions

Perinatal clinical characteristics included maternal age, assisted conception, maternal diabetes, maternal hypertension, clinical chorioamnionitis, preterm premature rupture of membranes (more than 24 h prior to delivery), delivery mode, prenatal antibiotics exposure, and singleton, gestational age (GA), birthweight (BW), sex, order of delivery (for multiples), and APGAR score at 5 min, abnormal temperature at admission, respiratory status, response to noxious stimuli, diagnosis of respiratory distress syndrome, pneumothorax, and early onset sepsis (EOS). The definitions of the above perinatal clinical characteristics were described in Supplemental materials.

### Outcomes

sIVH was defined as greater than or equal to grade 3 according to the Papile criteria [2]. The non-sIVH group was defined as infants without IVH or grade 1 or grade 2 IVH according to the Papile criteria [2].

### Standardization of early perinatal practices across the multiple neonatal intensive care units

To compare the practices across the multiple NICUs, we standardized each practice according to the perinatal clinical characteristics associated sIVH (SupFig. 1). We first calculated the expected probabilities of each practice. The expected probabilities were obtained using logistic regression models including a priori identified confounders for each practice. We reviewed the European Consensus Guidelines on the management of respiratory distress syndrome (2022 update) [28] and discussed each practice with experienced neonatologists to identify the confounders (see detail in the Supplementary materials). Among the studied practices, the antenatal corticosteroids, antenatal MgSO4 therapy, and caffeine treatment are the most evidence-based practice for VPIs currently, therefore, the expected probabilities for these three practices are 100%. In this study we did not account that the placenta transfusion was the most evidence-based practice, because the placenta transfusion was defined as receiving delayed cord clamping or cord milking. Delayed cord clamping is beneficial for VPIs [29], while the evidence of the cording milking for reducing IVH in VPIs is lacking [30]. The European guideline suggested that when delayed cord clamping is not feasible, consider umbilical cord milking in infants with GA more than 28 weeks [28]. Moreover, a noninferiority randomized controlled trial concluded that there was no difference in the rates of severe IVH between the umbilical cord milking versus delayed cord clamping in preterm infants born 28 to 32 weeks [31]. Therefore, we consider both in the practice of placenta transfusion. The expected model of inotropes given is difficult to build logistic regression models based on the limited information from the CHNN database (SupFigure 1 and SupFigure 2). Thus we could not standardize the practice of the inotropes given.

### Clustering the standardized practices

Furthermore, we investigated the patterns of these standardized practices associated with sIVH. We used ten practices with the exception of inotropes given for clustering these standardized practices (SupFigure 1 and SupFigure 2). The K-means algorithm was used to cluster these standardized practices into K-distinct clusters and Silhouette analysis was used to identify the best K value.

### Statistical analysis

Continuous variables were expressed as mean with SD, and categorical variables were expressed as numbers and percentages. Comparisons of clinical factors were performed by using the Welch’s t test or Wilcoxon rank sum test for continuous variables and the Chi-squared test or Fisher’s exact probability test for categorical variables. *P* < 0.05 was considered statistically significant. The odds ratios (ORs) and 95% confidence intervals were estimated in the multiple logistic regression. We performed all analyses using R software (version 4.0.3).

## Result

### Perinatal clinical factors among the enrolled very preterm infants with or without sIVH

The study cohort consisted of 24,226 VPIs from 55 participating NICUs with a mean (SD) GA of 29.5 (1.76) weeks and a mean (SD) BW of 1.31(0.32) kg. The rate of sIVH was 5.1% (1,231/24,226) (Table 1 and SupTable 1). A higher proportion of VPIs in sIVH group were male (62.6% vs 57.9%), lower BW (1.25[0.33] vs 1.31[0.32]), and lower GA (29.04[1.94] vs 29.53 [1.74]), and had APGAR score < 5 at five minutes (5.6% vs 3.3%).

**Table 1:** The perinatal clinical characteristics for the enrolled very preterm infants

Variables	Total *N* = 24,226	sIVH *N* = 1,231	Non-sIVH *N* = 22,995	*p*-value
**Prenatal period**				
Maternal age > 35 years, no (%)	5208 (21.5)	4948 (21.5)	4948 (21.5)	0.741
Assisted conception, no (%)	5814 (24.0)	313 (25.4)	5501 (23.9)	0.229
Diabetes, no (%)	5224 (21.6)	201 (16.3)	5023 (21.8)	< 0.001
Hypertension, no (%)	4463 (18.4)	274 (22.3)	4189 (18.2)	< 0.001
Clinical chorioamnionitis, no (%)	5183 (21.4)	244 (19.8)	4939 (21.5)	0.167
PROM, no (%)	5558 (22.9)	251 (20.4)	5307 (23.1)	0.029
Prenatal antibiotics exposure, no (%)	11710 (48.3)	604 (49.1)	11106 (48.3)	0.599
**At birth**				
Gestational age(wks), mean (SD)	29.50 (1.76)	29.04 (1.94)	29.53 (1.74)	< 0.001
Birthweight(kg), mean (SD)	1.31 (0.32)	1.25 (0.33)	1.31 (0.32)	< 0.001
Sex (male), no (%)	14085 (58.1)	771 (62.6)	13314 (57.9)	0.001
Delivery mode (cesarean), no (%)	14252 (58.8)	695 (56.5)	13557 (59.0)	0.083
Singleton/multiple births	13236/10990	719/512	12517/10478	0.006
Oder of delivery (non-first), no (%)	5694 (23.5)	295 (24.0)	5399 (23.5)	0.696
Apgar 5 score < 5, no (%)	820 (3.4)	69 (5.6)	751 (3.3)	< 0.001
**During hospitalization within 3 DOL**				
Abnormal temperature at admission, no (%)	4549 (18.8)	267 (21.7)	4282 (18.6)	0.007
Respiratory status(severe), no (%)	7002 (28.9)	663 (53.9)	6339 (27.6)	< 0.001
Inappropriate response to noxious stimuli, no (%)	549 (2.3)	116 (9.4)	433 (1.9)	< 0.001
RDS, no (%)	13511 (55.8)	987 (80.2)	12524 (54.5)	< 0.001
Pneumothorax, no (%)	296 (1.2)	90 (7.3)	206 (0.9)	< 0.001
EOS, no (%)	333 (1.4)	32 (2.6)	301 (1.3)	< 0.001
**Outcomes**				
Death	2026 (8.4)	235 (19.0)	1791 (7.8)	< 0.001
DAMA	2408 (9.9)	214 (17.0)	2194 (9.5)	< 0.001

*sIVH* severe intraventricular hemorrhage, *DAMA* discharge against medical advice, *EOS* early onset sepsis, *DOL* day of life

### Characterizing perinatal practices among the enrolled very preterm infants

**Fig. 2:**
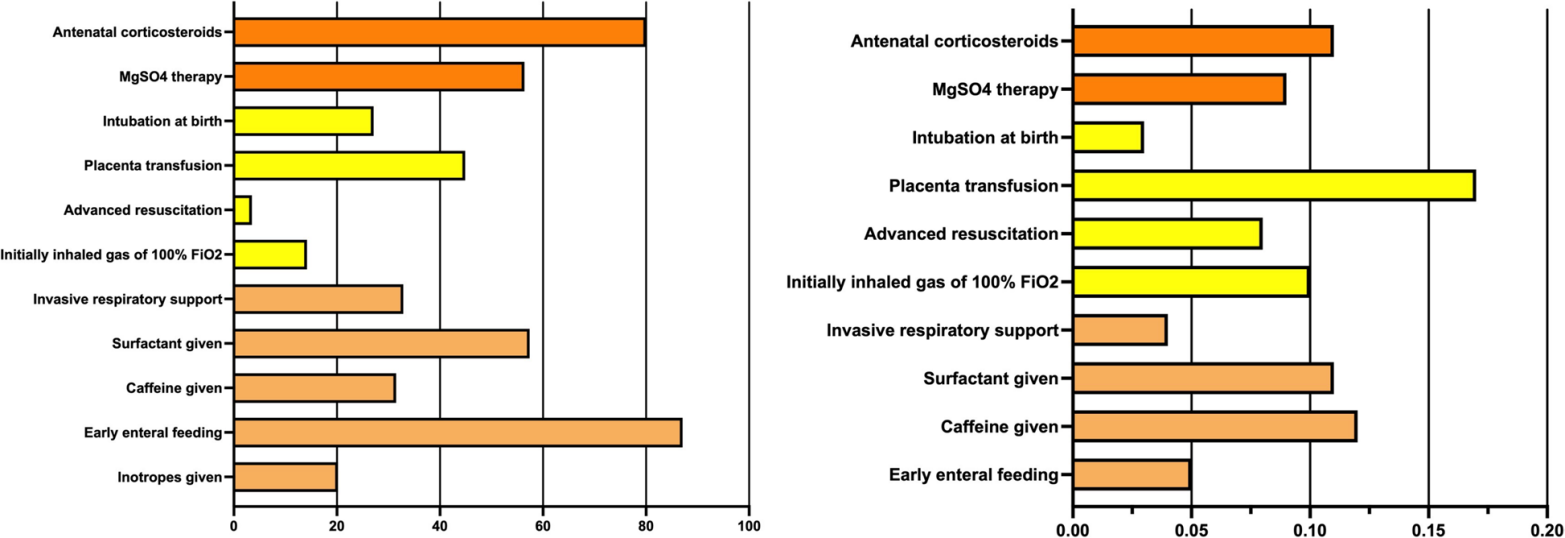
The performances of the early care practices among the enrolled very preterm infants. **A** The percentage of utility of the care practices among the enrolled very preterm infants. **B** The standard deviation of the performance of the care practices among the enrolled very preterm infants. The higher SD meant higher variation. The expected model of inotropes given is difficult to build logistic regression models based on the limited information from the CHNN database. Therefore, we could not standardize the practice of the inotropes given

Regarding the eleven practices (Fig. 2A), 80.0% (19,389/24,226) of the VPIs received antenatal corticosteroids, 56.4% (13,672/24,226) had antenatal MgSO4 therapy, 44.9% (10,868/24,226) had placental transfusion, and 31.5% (7,623/24,226) received caffeine within 3 DOL. After standardizing the performance of each practice across the multiple NICUs, the largest variation among the eleven practices was the practice of the placental transfusion (SD 0.17) (Fig. 2B).

### Practices associated with severe intraventricular hemorrhage in this cohort

**Fig. 3:**
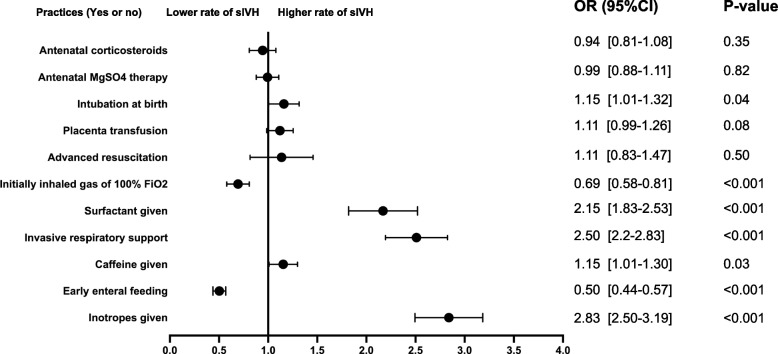
The risk-adjusted odds ratios of the care practices for severe intraventricular hemorrhage after adjustment for the perinatal clinical characteristics. The regressions of the antenatal corticosteroids and MgSO4 therapy controlled for birthweight, gestational age, Apgar 5 score less than 5, EOS, inotropes given, invasive respiratory support; The regressions of the intubation at birth, placenta transfusion, advanced resuscitation, initially inhaled gas of 100% FiO2 controlled for birthweight, gestational age, EOS, inotropes given, Apgar 5 score less than 5, invasive respiratory support; The regressions of the invasive respiratory support controlled for birthweight, gestational age, Apgar 5 score less than 5, EOS, inotropes given; The regressions of the caffeine given controlled for birthweight, gestational age, EOS, inotropes given, Apgar 5 score less than 5, invasive respiratory support; The regression of the early enteral feeding controlled for birthweight, gestational age, EOS, inotropes given, Apgar 5 score less than 5, invasive respiratory support; The regression of the inotropes given controlled for birthweight, gestational age, EOS, Apgar 5 score less than 5, invasive respiratory support. The regression of the surfactant given controlled for birthweight, gestational age, EOS, Apgar 5 score less than 5, inotropes given, invasive respiratory support

In this large cohort, we investigated the practices associated with sIVH via multiple logistic regression. Compared to the VPIs with non-sIVH, the VPIs with sIVH were more likely to be intubated at birth (AOR 1.15, 95% CI 1.01 to 1.32), receive initial invasive respiratory support (AOR 2.50, 95% CI 2.20 to 2.83), require surfactant (AOR 2.15, 95% CI 1.83 to 2.53), given caffeine (AOR 1.15, 95% CI 1.01 to 1.30) and inotropes (AOR 2.83, 95% CI 2.50 to 3.19). While, infantes with early enteral feeding (AOR 0.50, 95% CI 0.44 to 0.57) or initially inhaled gas of 100% FiO2 (AOR 0.69, 95% CI 0.58 to 0.81) less likely diagnosed with sIVH (Fig. 3).

### Identifying two clusters of the practices in NICU level

**Fig. 4:**
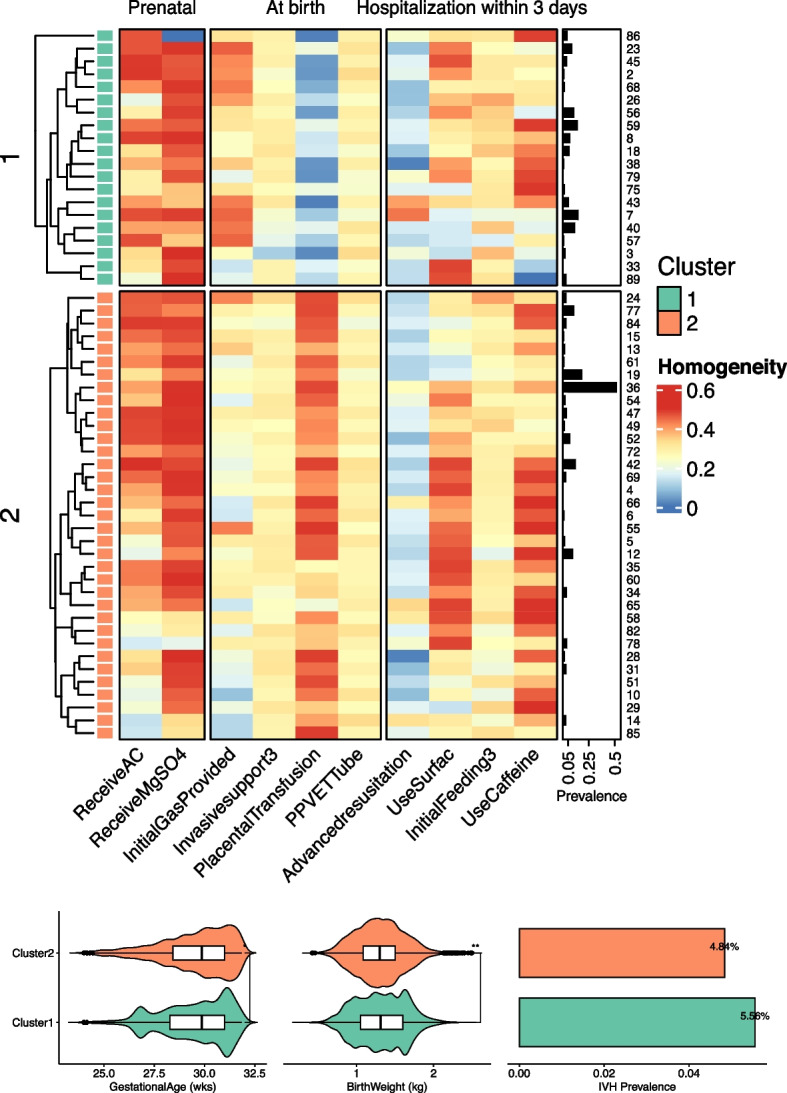
The clusters of the practices and the outcomes among the different clusters. **A** The two clusters of the practices. **B** The occurrence of sIVH among the two clusters

According to the standardizing practices, there were the significant practice variations across the 55 NICUs (SupFigure 3). We further clustered these practices, and two clusters were identified (k = 2) (SupFigure 4), including 8,204 VPIs from 20 NICUs, and 16,022 VPIs from 35 NICUs in the cluster 1, and cluster 2, respectively (Fig. 4A, and SupTable 2).

Cluster 2 had the highest use of antenatal MgSO4 therapy (60.8%), placenta transfusion (52.0%), caffeine given within 3 DOL (32.2%), and enteral feeding within 3 DOL (89.5%); but the lowest rates of initial inhaled gas of 100% FiO2 (8.9%), advanced resuscitation (3.0%), intubation at birth (24.8%), invasive ventilation support within 3 DOL (28.2%), surfactant given (54.6%) and inotropes give (17.3%) (SupTable 2).

Regarding the variations of the practices among the two clusters (SupFigure 3), the cluster 2 showed lowest variation in the initial inhaled gas of 100% FiO2 (SD = 0.238, *p* < 0.05), but higher variation in the placenta transfusion (SD = 0.421, *p* < 0.05), and caffeine given (SD = 0.393, *p* < 0.05). The variations of the remaining practices were not significantly different among the two clusters.

Regarding the outcomes (Fig. 4B), the lowest rate of sIVH (4.8%) was observed in the cluster 2 (SupTable 2). Comparing to the perinatal clinical characteristics in the cluster 1, the VPIs in the cluster 2 were the higher GA but lower BW. Furthermore, we compared the sIVH of VPIs among the two clusters after stratifying two major perinatal clinical characteristics including gestational age, and early onset sepsis (SupTable 2). We observed that the rate of sIVH was still the lower (4.2%) in cluster 2 among the VPIs with GA of more than 28 weeks but higher among the VPIs with GA of less than 28 weeks (SupTable 3). While, the rate of the sIVH was consistently lower in the cluster 2 among the VPIs with EOS (SupTable 4).

## Discussion

In this large VPIs cohort from the multiple NICUs, we found that 5.1% of VPIs had sIVH. The occurrence of sIVH in this cohort was not high possibly due to the higher gestational age admitted to NICUs in the CHNN database.

China has a vast territory and with great heterogeneity of population and medical care. Therefore, we standardized the studied practices to compare the performance of these practices across the multiple NICUs from China. Among these practices, we observed that the antenatal corticosteroids and MgSO4 therapy were not performed in each VPI as expected. We also observed that most NICUs started enteral feeding within 3 DOL. Regarding the other beneficial practice of caffeine administration, the percentage of this practice was not high and the variation of this practice was large. This finding indicated that the caffeine given within 3 DOL was not routine practice in VPIs of the Chinese NICUs.

The percentage of the utility of the advanced resuscitation was lowest, which could suggest the improvement of the resuscitation for VPIs. However, the percentages of the utilities of the invasive respiratory support and intubation at birth were relatively low, indicating that the invasive procedure tended to be limited in Chinese NICU currently.

Studies suggest that optimal care practices [32] or the application of neonatal care bundles [18] could significantly reduce risk of developing sIVH and the critical care time was from the perinatal period to the first 3 DOL [27]. However, there are few studies to systemically investigate the associations between the practices and the occurrences of sIVH in detail. Different from the study [33], we did not find the practices of the antenatal corticosteroids and MgSO4 therapy were associated with the lower rate of sIVH. However, another systematic review indicated that although the antenatal MgSO4 could decreased the rate of IVH in preterm infant, this effect was not statistically significant. This could be related to the dosage, timing and gestational age [34]. In our study, our primary outcome was sIVH other than IVH. Moreover, the other invasive practices could contribute more to the occurrence of sIVH in our cohort. We addressed intubation at birth, and within 3 DOL the practices of: invasive respiratory support, caffeine administration, inotropes use, and enteral feeding. Among the studied care practices, the invasive practices (intubation at birth, invasive respiratory support, surfactant given) and inotropes use were risk factors for sIVH. This finding is consistent with other studies [35, 36]. The study suggests that the underlying pathophysiology of invasive practices may be related to the inflammatory and hemodynamic pathway [37]. Interestingly [38], caffeine given within 3 DOL was risk factor of sIVH in our study, however the OR was close to 1. Previous studies indicated that caffeine given on day 1 or day 2 was a protective factor of preterm brain injury (sIVH, cystic periventricular leukomalacia, and posthaemorrhagic ventricular dilatation) [38]. Therefore, the possible reason could be that the caffeine was given to the VPIs with apnea caused by sIVH in China. Further analysis should be conducted on the associations between the time of caffeine given and sIVH.

Consist with the study [39], we found initiation of enteral feeding within 3 DOL was a protective factor for developing sIVH. Physiologically, studies showed that early feeding was associated with increased superior mesenteric artery blood flow, and decreased intestinal vascular resistance, which has uncertain association with the occurrence of sIVH [40, 41].

Regarding the variations of the practices among the two clusters, a lower variation meant that the observation was closer to the expected probability, which indicated that the neonatologists could perform the practices as the newborns required. However, the absolute values for the difference between expected and observed probabilities cannot give more information regarding whether these interventions were unnecessarily performed or omitted when necessary. In our results, we found that the practice of placental transfusion showed higher deviation in the cluster 2 where the incidence of sIVH was low, while the rate of performance of placental transfusion in the cluster 2 NICUs (52.0%) was higher than that in the cluster 1(30.9%). These could be explained that this high variability could potentially lead to a reduced incidence of sIVH. This might also indicate that the performance of the placental transfusion should depend on the neonates’ current conditions and physicians’ personal skills in practice.

Furthermore, we observed two clusters of the care practices amongst multiple NICUs. Among the two clusters, the practice in the cluster 2 was consistent with target practices as described in quality improvements on IVH [32]. Comparing the perinatal clinical characteristics and outcomes among these clusters, we observed that the sIVH was lower in the cluster 2, even though the cluster 2 had the more VPIs with risk profiles (such as EOS). These findings suggest closer adherence to best practices could reduce the occurrence of sIVH.

### Limitations

The rate of sIVH was as high as 50% in some NICUs, therefore, some NICUs could not upload the cases completely. We extracted the practices within 3 DOL, such as caffeine use, inotropes given, and invasive ventilation. However, the sIVH could occur before these practices are performed. More evidence has showed that the prophylaxis with intravenous indomethacin in extremely low birth weight infants may reduce sIVH. However, there was only 3 infants with intravenous indomethacin in our cohort. Lastly, we did not consider the quality of interventions in each NICU because it is difficult to be estimated based on the current dataset. We also adjusted for as many confounders as possible, but we likely were not able to adjust for all.

## Conclusion

The current evidence-based practices including the antenatal corticosteroids, MgSO4 therapy, and caffeine given within 3 DOL were not performed in each VPI as expected. Moreover, there were practical variations across multiple NICUs in China. In this large VPIs cohort study, invasive practice, inotropes given may increase the risk of sIVH, while, starting the enteral feeding early may reduce the risk of sIVH. These findings would help to better understand the current status of the care practices for VPIs in China, and target further quality improvement initiatives on reducing sIVH in Chinese NICUs.

### Supplementary Information

**Supplementary Material 1.**

## Data Availability

Data are available to the corresponding author upon a reasonable request.

## Abbreviations

NICUs: Neonatal intensive care units
IVH: Intraventricular hemorrhage
sIVH: Severe intraventricular hemorrhage
VPIs: Very preterm infants
CHNN: Chinese Neonatal Network
DAMA: Discharge against medical advice
DOL: Days of life
MgSO4: Antenatal magnesium sulphate
GA: Gestational age
BW: Birthweight
EOS: Early onset sepsis
ORs: Odds ratios
