# Supplementary material for: The performance of the practices associated with the occurrence of severe intraventricular hemorrhage in the very premature infants: data analysis from the Chinese neonatal network
Source: BMC Pediatr. 2024 Jun 14;24:394. doi: 10.1186/s12887-024-04664-8 (PMC11179376; doi:10.1186/s12887-024-04664-8)
Supplement: Supplementary file 1 — Supplementary Material 1. [file 12887_2024_4664_MOESM1_ESM.docx]

**Methods**

**Data collection and datasets**

Trained data abstractors were responsible for data acquisition in each hospital. Data is directly entered into a customized database with built-in error checking and a standard manual of operations and definitions. Data is then electronically transmitted to the CHNN coordinating center in the Children’s Hospital of Fudan University with records de-identified. Site investigators were responsible for data quality control at each site.

**The definition of the perinatal practice and clinical characteristics**

Antenatal corticosteroid was defined as a partial or complete course of antenatal corticosteroids before birth. Advanced resuscitation was defined as requiring chest compression of more than 30 seconds and/or bolus epinephrine use. Placenta transfusion was defined as receiving delayed cord clamping or cord milking. Enteral feeding type is defined by whether the infants were fed breast milk or formula or donor milk within 3 DOL. The use of inotropes included use of any or combination of dopamine, dobutamine, epinephrine, norepinephrine, and milrinone infusions.

GA was determined using (in order of preference based on availability) prenatal ultrasonography, menstrual history, obstetric examination, or all three methods of evaluation. If the obstetric estimate was not available or was different from the postnatal pediatric estimate of gestation by more than two weeks, the gestational age was estimated using the Ballard Score. Clinical chorioamnionitis was defined as either clinically suspected and/or pathogenic diagnoses. Respiratory distress syndrome was defined as neonates with clinical and radiographic evidence of respiratory distress syndrome and requiring surfactant therapy. EOS was defined by the presence of clinical symptoms and a positive culture from blood or cerebrospinal fluid samples drawn within 3 DOL. Abnormal temperature at admission was defined as the temperature less than 36.0 ˚ C or more than 38.0 ˚ C. Respiratory status was defined as severe (apnea, gasping, intubated) and non-severe referring to transport risk index of physiologic stability, version II (TRIPS-II)^1^. Response to noxious stimuli was defined as inappropriate (none, seizure, muscle relaxant, lethargic response, no cry) and appropriate referring to TRIPS-II^1^.The respiratory status and response to noxious stimuli referring to TRIPS-II were assessed when the neonates were admitted in NICU.

**Standardization of early perinatal practices across the multiple neonatal intensive care units**

We reviewed the European Consensus Guidelines on the management of respiratory distress syndrome (2022 update) and discussed each practice with experienced neonatologists^2^. Except that antenatal corticosteroids, antenatal MgSO4 therapy and caffeine are highly recommended in very preterm infants based on current guideline, other practices related to sIVH were performed according to the clinical condition. The intubation at birth, placenta transfusion, initially inhaled 100% FiO2, and advanced resuscitation are performed in the delivery room. The decision for these practices highly depended on the newborns’ condition. Apgar score is an optimal and available variable from the CHNN dataset to assess the newborns’ condition in the delivery room. Commonly, the delivery type could be an indicator reflecting the mother’s condition, and also it could affect the practice of placenta transfusion. The invasive respiratory support, surfactant given, and early feeding were performed in NICU. The decision for these practices mainly depended on the gestational age and birthweight. However, for the invasive respiratory support, the newborns’ situation (Apgar score) is another important variable. The current recommendation for surfactant given should be consider whether the VPIs require intubation for stabilization and the level of respiratory support. Therefore, the diagnosis of respiratory distress syndrome (RDS) is an optimal and available variable from the CHNN dataset to assess the VPIs’ situation.

**Results**

**SupTable1. The number of sIVH across different gestational age in our cohort.**

| Weeks | Number of sIVH (n, %) |
| --- | --- |
| <=24+6 (n= 339） | 38 (11.2) |
| 25（n= 629） | 46 (7.3) |
| 26（n= 1661） | 125 (7.5) |
| 27（n= 1945） | 117 (6.0) |
| 28（n= 3404） | 189 (7.3) |
| 29（n= 4263） | 268 (6.3) |
| 30（n= 5417） | 200 (3.7) |
| 31（n= 6568） | 248 (3.8) |

**SupTable 2. The characteristics of early care practices among the two clusters**

| Variables | Cluster 1  N = 8,204  (20 NICUs) | Cluster 2  N = 16,022  (35 NICUs) | P-value |
| --- | --- | --- | --- |
| Prenatal period |  |  |  |
| Antenatal corticosteroids, Yes (%) | 6,531 (79.6) | 12,858 (80.3) | 0.235 |
| MgSO4 therapy, Yes (%) | 3,938 (48.0) | 9,734 (60.8) | <0.001 |
| At delivery room |  |  |  |
| Intubation at birth, Yes (%) | 2,598 (31.7) | 3,968 (24.8) | <0.001 |
| Placenta transfusion, Yes (%) | 2,533 (30.9) | 8,335 (52.0) | <0.001 |
| Advanced resuscitation, Yes (%) | 373 (4.5) | 484 (3.0) | <0.001 |
| Initially inhaled gas of 100% FiO2 (%) | 2,012 (24.5) | 1,430 (8.9) | <0.001 |
| Hospitalization within 3 DOL |  |  |  |
| Invasive respiratory support, Yes (%) | 3,456 (42.1) | 4,511 (28.2) | <0.001 |
| Surfactant given, Yes (%) | 5,167 (63.0) | 8,748 (54.6) | <0.001 |
| Caffeine given, Yes (%) | 2,469 (30.1) | 5,154 (32.2) | <0.001 |
| Early enteral feeding, Yes (%) | 6,764 (90.6) | 14,335 (89.5) | <0.001 |
| Inotropes given, Yes (%) | 2,121 (25.9) | 2,775 (17.3) | <0.001 |

sIVH: severe intraventricular hemorrhage

**SupTable 3. The perinatal clinical characteristics among the two clusters of the practices**

| Variables | Cluster 1  N = 8,204  (20 NICUs) | Cluster 2  N = 16,022  (35 NICUs) | P-value |
| --- | --- | --- | --- |
| Prenatal period |  |  |  |
| Maternal age>35 years, n (%) | 1,674 (20.4) | 3,534 (22.1) | 0.003 |
| Assisted conception, n (%) | 1,800 (21.9) | 4,014 (25.1) | <0.001 |
| Diabetes, n (%) | 1,838 (22.4) | 3,386 (21.1) | 0.023 |
| Hypertension, n (%) | 1,318 (16.1) | 3,145 (19.6) | <0.001 |
| Clinical chorioamnionitis, n (%) | 2,157 (26.3) | 3,026 (18.9) | <0.001 |
| PROM, n (%) | 1,755 (21.4) | 3,803 (23.7) | <0.001 |
| Prenatal antibiotics exposure, n (%) | 3,446 (42.0) | 8,264 (51.6) | <0.001 |
| At birth |  |  |  |
| Gestational age (weeks), mean (SD) | 29.47 (1.72) | 29.52 (1.78) | 0.002 |
| Birthweight (kilogram), mean (SD) | 1.32 (0.33) | 1.30 (0.31) | <0.001 |
| Sex (male), n (%) | 5,080 (61.9) | 9,005 (56.2) | <0.001 |
| Delivery mode (cesarean), n (%) | 4,596 (56.0) | 9,656 (60.3) | <0.001 |
| Singleton/multiple births | 4,532/3,672 | 8,704/7,318 | 0.175 |
| Oder of delivery (non-first), n (%) | 2,058 (25.1) | 3,636 (22.7) | <0.001 |
| Apgar 5 score < 5, n (%) | 369 (4.5) | 451 (2.8) | <0.001 |
| During hospitalization within 3 DOL |  |  |  |
| Abnormal temperature at admission, n (%) | 2,125 (25.9) | 2,424 (15.1) | <0.001 |
| Respiratory status(severe), n (%) | 2,346 (28.6) | 4,656 (29.1) | 0.451 |
| Inappropriate response to noxious stimuli, n (%) | 212 (2.6) | 337 (2.1) | 0.017 |
| RDS, n (%) | 5,030 (61.3) | 8,481 (52.9) | <0.001 |
| Pneumothorax, n (%) | 96 (1.2) | 200 (1.2) | 0.600 |
| EOS, n (%) | 94 (1.1) | 239 (1.5) | 0.029 |
| Outcomes |  |  |  |
| sIVH | 456 (5.6) | 775 (4.8) | 0.016 |

sIVH: severe intraventricular hemorrhage, DAMA: discharge against medical advice, EOS: early onset sepsis.

**SupTable 4. Comparison of the occurrences of sIVH among the two clusters after stratifying the gestational age**

|  | sIVH | sIVH in VPIs with GA  < 28 weeks  (n, %) | sIVH in VPIs with GA  ≥ 28 weeks  (n, %) |
| --- | --- | --- | --- |
| Cluster 1  N=8,204 | 456 | 107/1,675 (6.4) | 349/6,529 (5.3) |
| Cluster 2  N=16,022 | 775 | 219/2,899 (7.6) | 556/13,123 (4.2) |

**SupTable 5. Comparison of the occurrences of sIVH among the two clusters after stratifying the diagnosis of sepsis**

|  | sIVH | sIVH in VPIs with EOS (n, %) | sIVH in VPIs without EOS (n, %) |
| --- | --- | --- | --- |
| Cluster 1  N=8,204 | 456 | 23/94 (24.5) | 433/8110 (5.3) |
| Cluster 2  N=16,022 | 775 | 9/239 (3.8) | 766/15,783 (3.7) |

SupFigure 1.


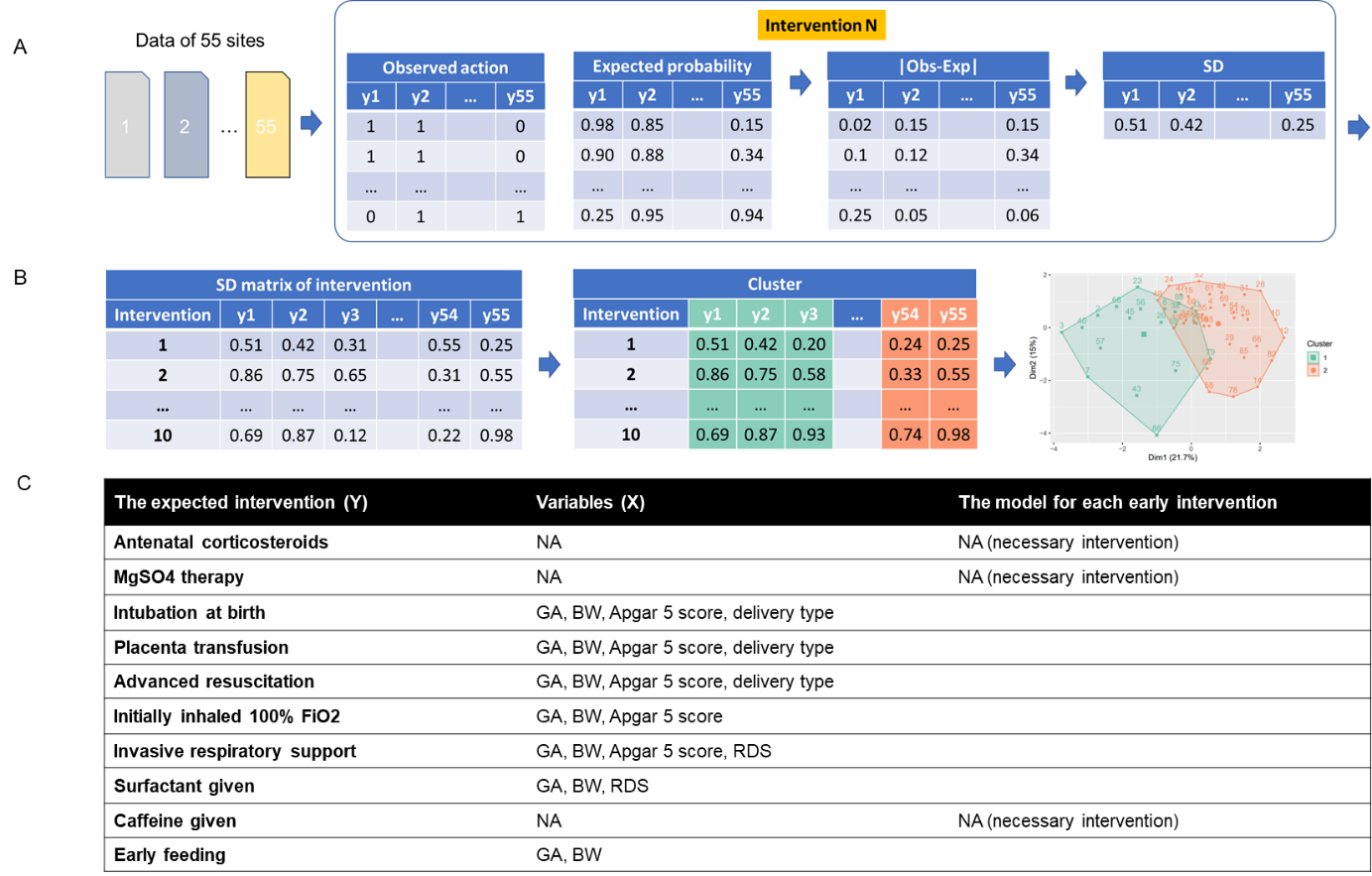


A. The process of the calculation of the standard deviations of distributions of expected probabilities and observations in each practice.

B. Clustering the enrolled NICUs based on the similar the standard deviations of distributions of expected probabilities and observations in each practice.

C. The adjusted variables for the prediction model of the expected probabilities in each practice. To predict the probabilities of expected practices, we built logistic regression models for each practice in each of the 25 hospitals with an average of >150 infants per year, choosing the center with the largest area under the receiver operating characteristic curve (AUC) score for each practice as the training sets of practice models. Using the practice of placenta transfusion as an example, we first calculated the expected probability of each infant receiving this practice. Then, each infant would have a difference value between the observed and the expected probability in this practice. The standard deviation (SD) of the difference values for all infants in each NICU was used as the variation of this practice. A lower variation meant that the observation was closer to the expected probability.

GA: gestational age, BW: birthweight, RDS: respiratory distress syndrome

SupFigure 2. The receiver operating characteristic curve for these prediction models


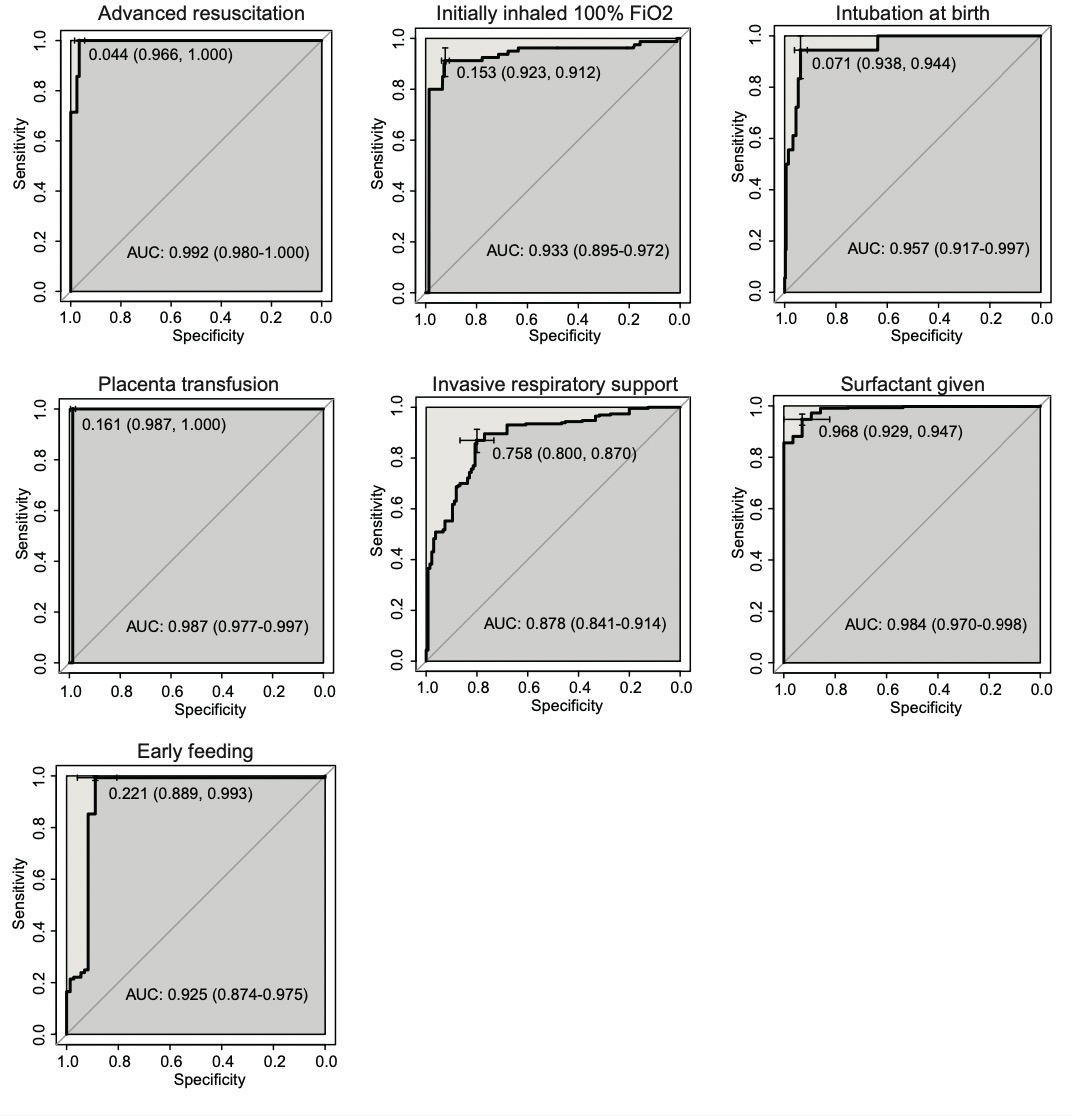


SupFigure 3. The variations of the care practices among the two clusters.


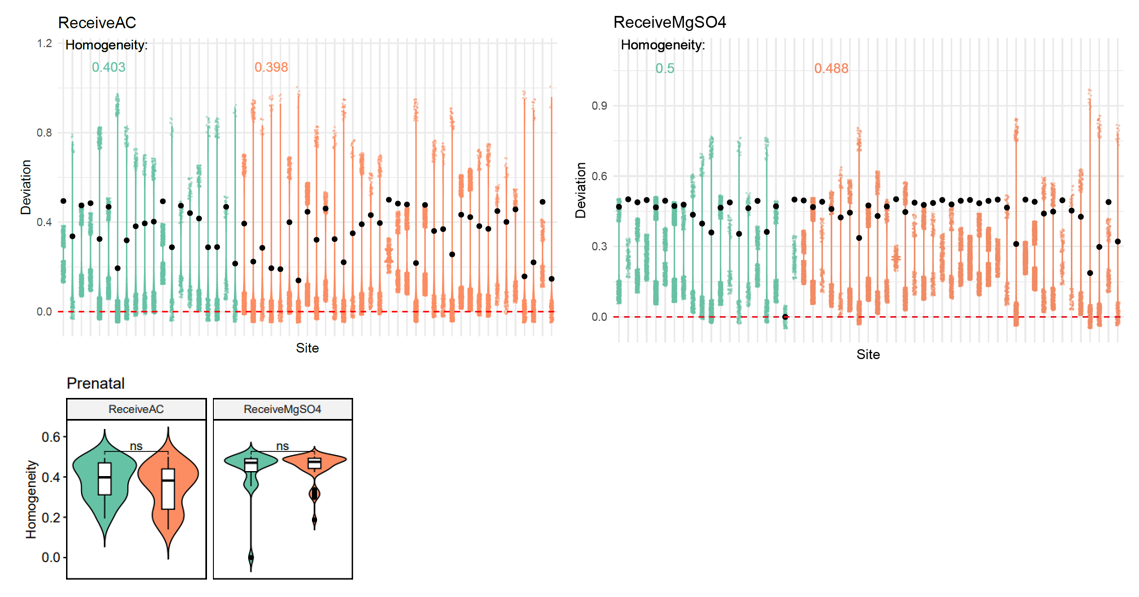

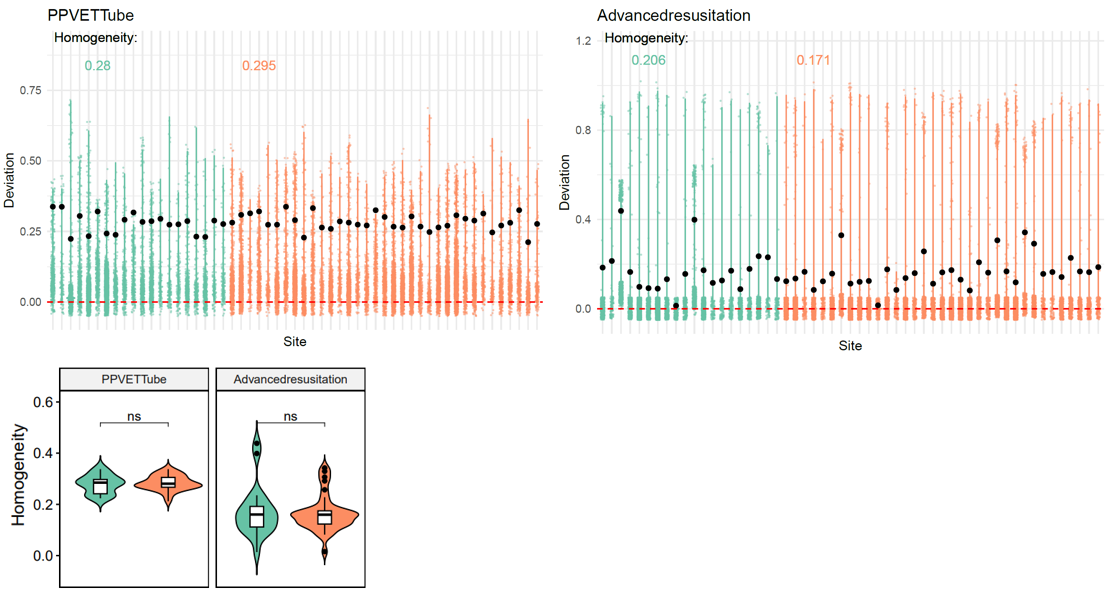


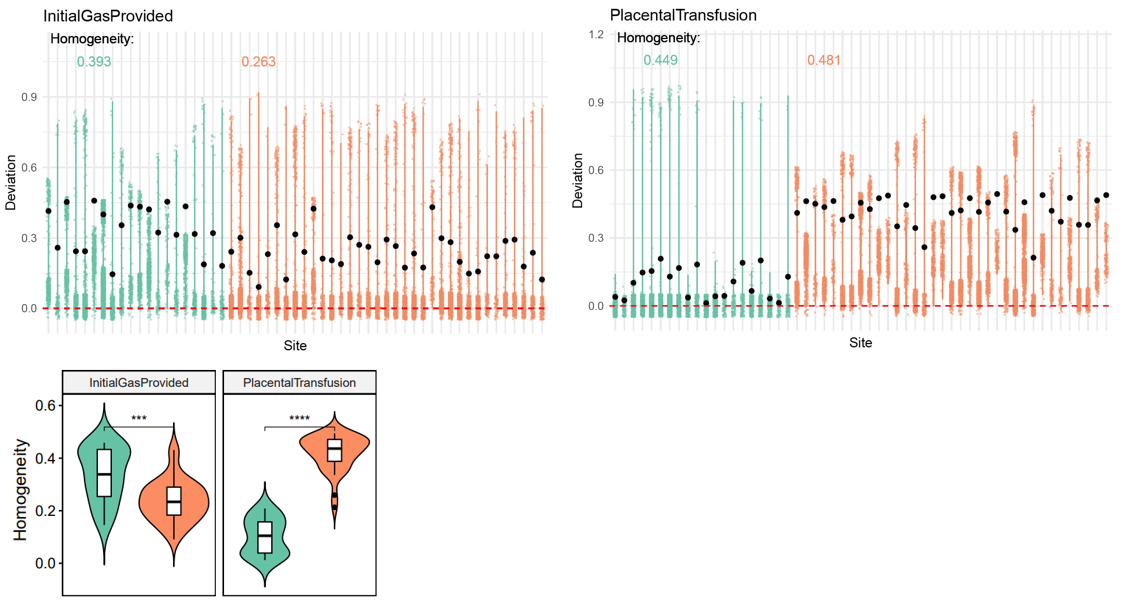

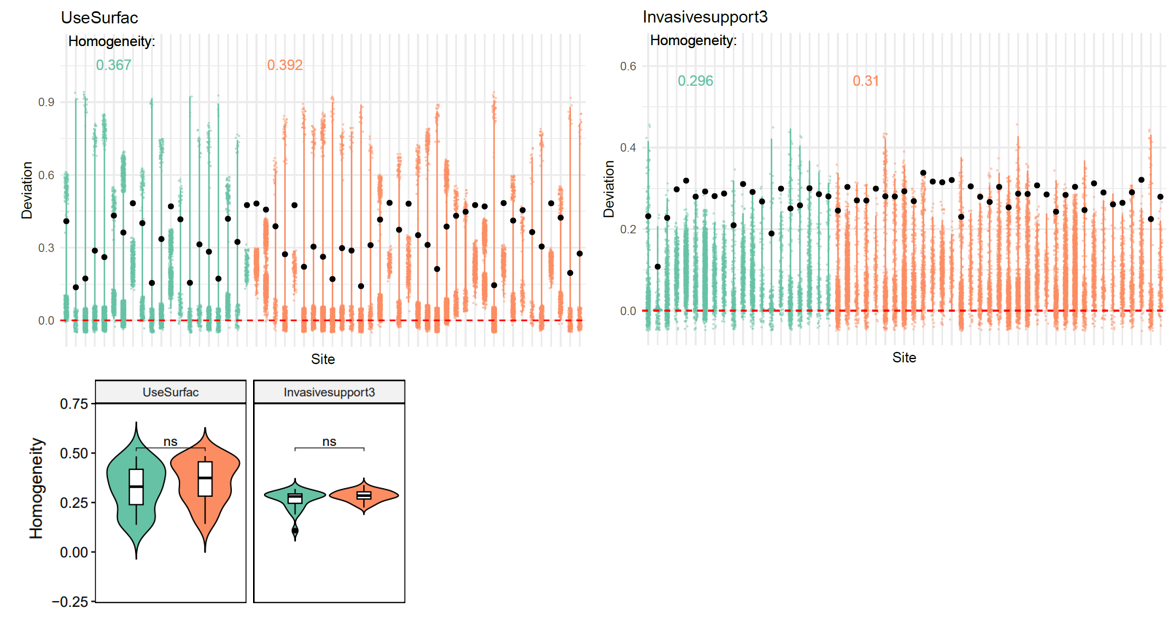


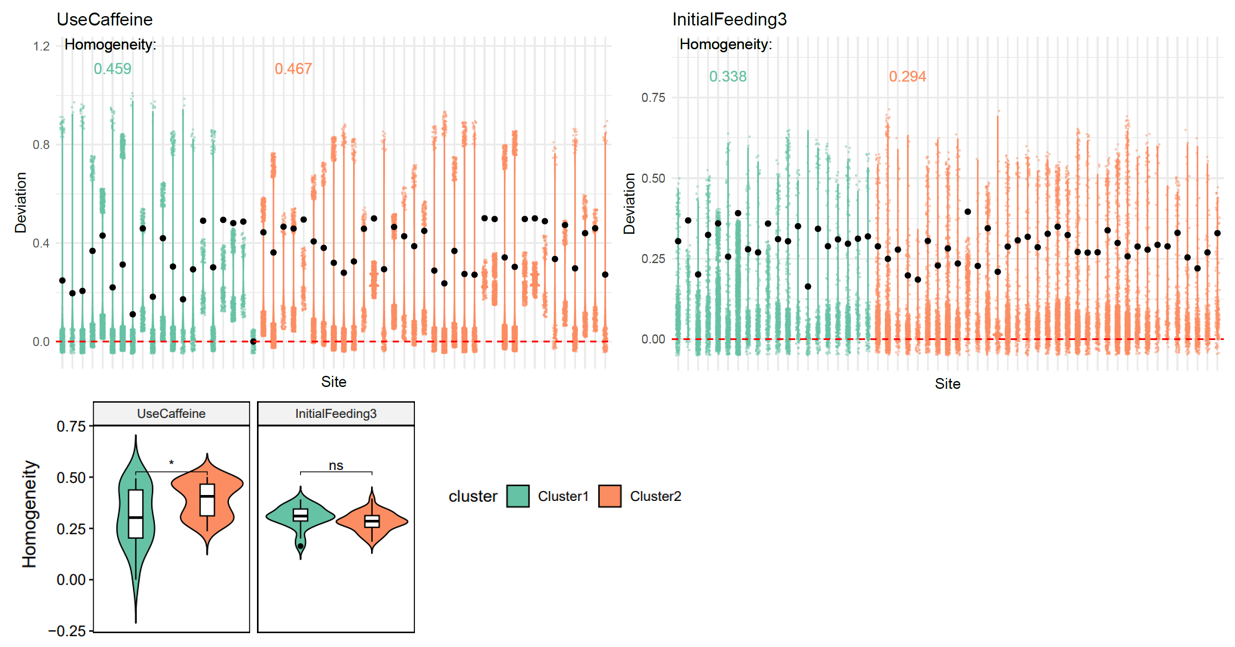


SupFigure 4. The K distinct clusters and Silhouette analysis


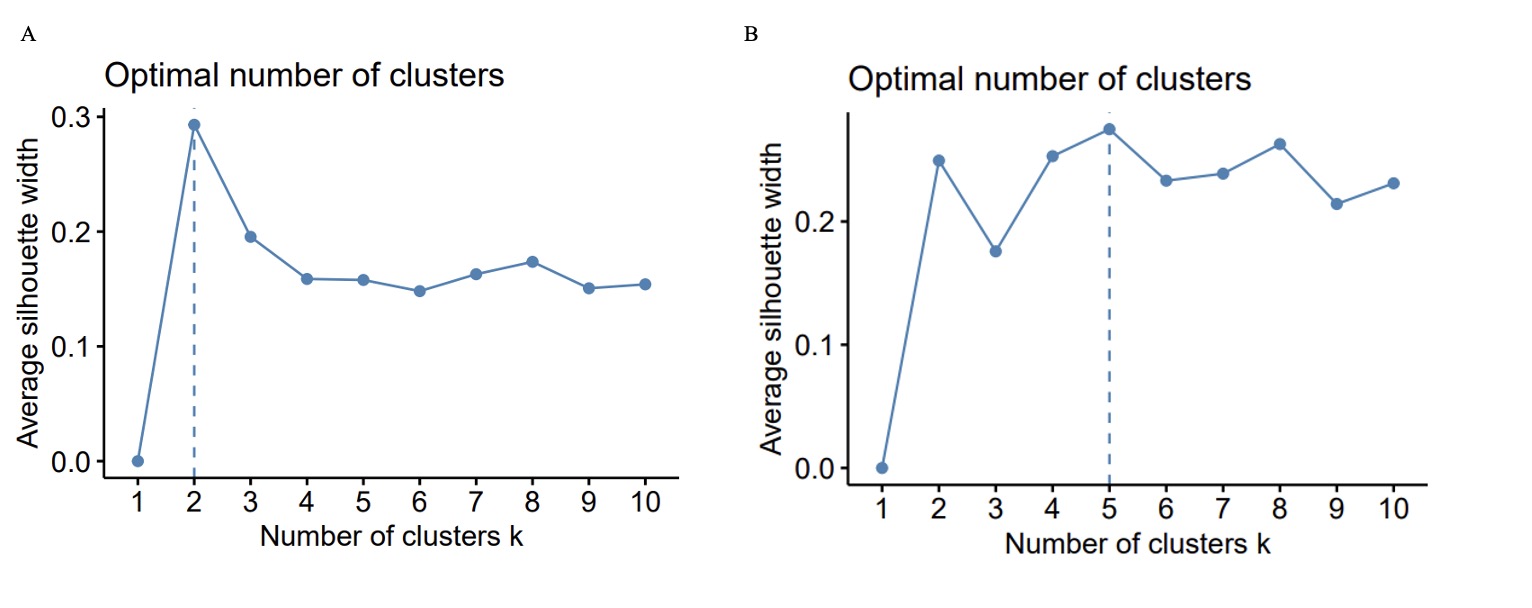


The K distinct clusters and Silhouette analysis.A. All 10 practices for NICU clustering (the value of Silhouette analysis 0.30), B: The identified 5 practices associated with sIVH through multivariable logistic regression for NICU clustering (the value of Silhouette analysis 0.27).

Reference

1. Lee SK, Aziz K, Dunn M, et al. Transport Risk Index of Physiologic Stability, version II (TRIPS-II): a simple and practical neonatal illness severity score. *Am J Perinatol.* 2013;30(5):395-400.

2. Sweet DG, Carnielli VP, Greisen G, et al. European Consensus Guidelines on the Management of Respiratory Distress Syndrome: 2022 Update. *Neonatology.* 2023;120(1):3-23.
